# Multiple additive moderation effects of insulin resistance and dietary inflammatory index on the relationship between body mass index and postprandial glucose levels in Korea: a retrospective analysis

**DOI:** 10.7586/jkbns.25.011

**Published:** 2025-05-16

**Authors:** Jiwon Park, Myoung Soo Kim

**Affiliations:** 1 School of Nursing, University of Maryland School of Nursing, Baltimore, USA; 2 Department of Nursing, Pukyong National University, Busan, Korea

**Keywords:** Body mass index, Diabetes mellitus, Diet, Glucose, Insulin resistance

## Abstract

**Purpose:**

This study examined the potential moderating effects of insulin resistance and dietary inflammatory index (DII) on the relationship between body mass index (BMI) and peak postprandial glucose levels in patients with prediabetes and type 2 diabetes.

**Methods:**

A retrospective analysis was conducted, using integrated nutritional data from a diabetes management program for patients with prediabetes and type 2 diabetes who used continuous glucose monitoring (CGM). Data from 17 patients, including 408 meal records, were analyzed. The energy-adjusted DII (E-DII) was calculated, and peak postprandial glucose was matched for each meal. Statistical tests analyzed differences and relationships between variables. Hayes' SPSS PROCESS macro version 4.0 was used to identify the moderating effects.

**Results:**

Peak postprandial glucose levels varied significantly among E-DII subgroups (*p* < .026) and were positively correlated with insulin resistance (rho = .30, *p* = < .001) and E-DII (rho = .14, *p* = .004). Insulin resistance and E-DII had a multiple additive moderating effect on the relationship between BMI and peak postprandial glucose levels in glucose-controlled patients (F = 12.22, *p* < .001), explaining 21.6% of the variance.

**Conclusion:**

Higher E-DII scores, which indicated a more pro-inflammatory diet, were associated with increased glucose levels, especially for patients who were overweight and mildly obese. Patients with obesity who received insulin therapy had varied postprandial glucose levels depending on their insulin resistance status. These findings highlight the importance of tailored dietary interventions, inflammation management, and CGM to improve obesity-related glucose regulation.

## INTRODUCTION

Diabetes has emerged as one of the biggest health problems threatening the global population, and the global prevalence is expected to reach 780 million by 2045 [1]. The prevalence of prediabetes and type 2 diabetes (T2D) among people in their 30s or older in Korea was 44.3% and 16.7%, respectively [2]. Over 24% of overweight patients or patients with obesity with diabetes perceived their weight to be normal and did not show weight loss effort [3]. Preventing progression from prediabetes to T2D requires intensive weight management and glucose control [4]. Effective glucose control involves managing glycated hemoglobin A1c (HbA1c), fasting blood glucose, and postprandial glucose levels; in particular, managing postprandial glucose levels plays a significant role in overall glucose control [5,6]. Postprandial hyperglycemia prevails among patients who are overweight or with obesity without diabetes, as indicated by a maximal continuous glucose monitoring (CGM) level of 200 mg/dL [7]. Postprandial glucose levels are positively correlated with body mass index (BMI) [8,9] and are influenced by insulin resistance and dietary inflammatory index (DII) [10,11].

Although interest in postprandial hyperglycemia has increased, there are limited studies on the relationships between BMI, insulin resistance, and DII. Recently, CGM has been shown to improve glucose control by identifying glucose variability, to improve dietary compliance, and to serve as biofeedback for patients with prediabetes or those who have undergone bariatric surgery [12]. CGM can be a useful tool for reinforcing behavioral interventions; however, there are several points to consider. For instance, although 87% of CGM users reported that they could make informed decisions about food choices [13], immediate feedback on dietary impacts on glucose regulation was reported to be essential [14]. The risk of decreased insulin sensitivity or reduced beta cell function does not change linearly with BMI [15]. It is necessary to investigate the role of insulin resistance caused by internal factors such as the number of insulin acceptors or intracellular defects and the role of DII, an external factor related to eating habits, to determine the relationship between BMI and postprandial hyperglycemia.

Postprandial glucose peaks 1 hour after starting a meal and then returns to previous levels within 2 to 3 hours [16]. Prolonged postprandial hyperglycemia contributes to diabetic complications [17]. Postprandial glucose has a more positive correlation with HbA1c than fasting blood glucose. According to other studies, the most important factors for postprandial glucose are BMI, insulin resistance, and dietary habits. Managing these variables for patients with obesity can prevent the progression of T2D in those at the prediabetic stage and vascular complications in those with diabetes [18,19].

Postprandial glucose in patients with obesity with a BMI of 30kg/m^2^ or higher was higher than those in the normal weight group for a long time [20]. There was a positive relationship between BMI and postprandial glucose [8], and BMI was a risk factor for postprandial glucose because a higher BMI was associated with higher postprandial hyperglycemia [9]. Thus, increased BMI causes prolonged postprandial hyperglycemia; therefore, proper management of obese-related indicators is important.

There are two main factors influencing postprandial hyperglycemia. Insulin resistance reduces the effectiveness of insulin, ultimately leading to hyperglycemia. When insulin resistance occurs along with a decrease in the ability of the pancreas to secrete insulin, postprandial glucose levels increase to an even greater extent [21]. Insulin resistance has been closely linked to several diseases such as obesity and T2D [22]. Hormones such as leptin and inflammatory cytokines increase insulin resistance, and adipocytes in individuals with obesity secrete higher quantities of these hormones [23,24]. Insulin resistance is significantly high, and the pancreas’ capacity to secrete insulin is significantly reduced in patients with prediabetes; the 1-hour postprandial glucose level is 155 mg/dL or higher [25]. Patients with prediabetes and diabetes with insulin resistance may experience prolonged hyperglycemia after eating, which can exacerbate diabetes-related indicators and metabolic diseases.

The DII was also positively associated with postprandial hyperglycemia. Changes in dietary habits can affect gut microbial activity and the immune system, leading to the potential for food to cause inflammation [26,27]. Shivappa et al. [28] proposed the DII to assess the inflammatory potential of a meal. Each nutrient and food are assigned an inflammatory effect score based on its impact on inflammatory cytokines and its pro-inflammatory potential. A previous study demonstrated a positive correlation between the DII and 2-hour plasma glucose concentrations after an oral glucose load [29]. The highest inflammatory diet group had a 3-fold higher risk of developing T2D compared to the lowest inflammatory diet group, even after adjusting for confounding variables [30]. Furthermore, there is more evidence of a positive relationship between a pro-inflammatory diet and an increased risk of developing T2D [30-33]. Therefore, this study examined the relationships between insulin resistance, DII scores, and postprandial glucose levels of patients with prediabetes and diabetes with CGM.

## METHODS

### 1. Study design

This study is a secondary analysis that evaluated the effectiveness of non-contact dietary coaching using a CGM device for adult patients with diabetes or prediabetes [34]. A randomized controlled trial to investigate the effectiveness of dietary coaching and CGM. After the intervention, men in the experimental group had improved thigh circumference, and women in the experimental group had improved eating self-efficacy.

### 2. Participants

Participants were recruited from two outpatient departments. Patients with diabetes mellitus receiving treatment at the Endocrinology Department and patients with prediabetes visiting an Obesity Clinic at Kosin University Gospel Hospital were included in this study. They wore a CGM, received individual dietary coaching 5 days a week, and participated in group coaching once a week for 4 weeks. The intervention was conducted from November 2020 to April 2021.

**Figure 1.**
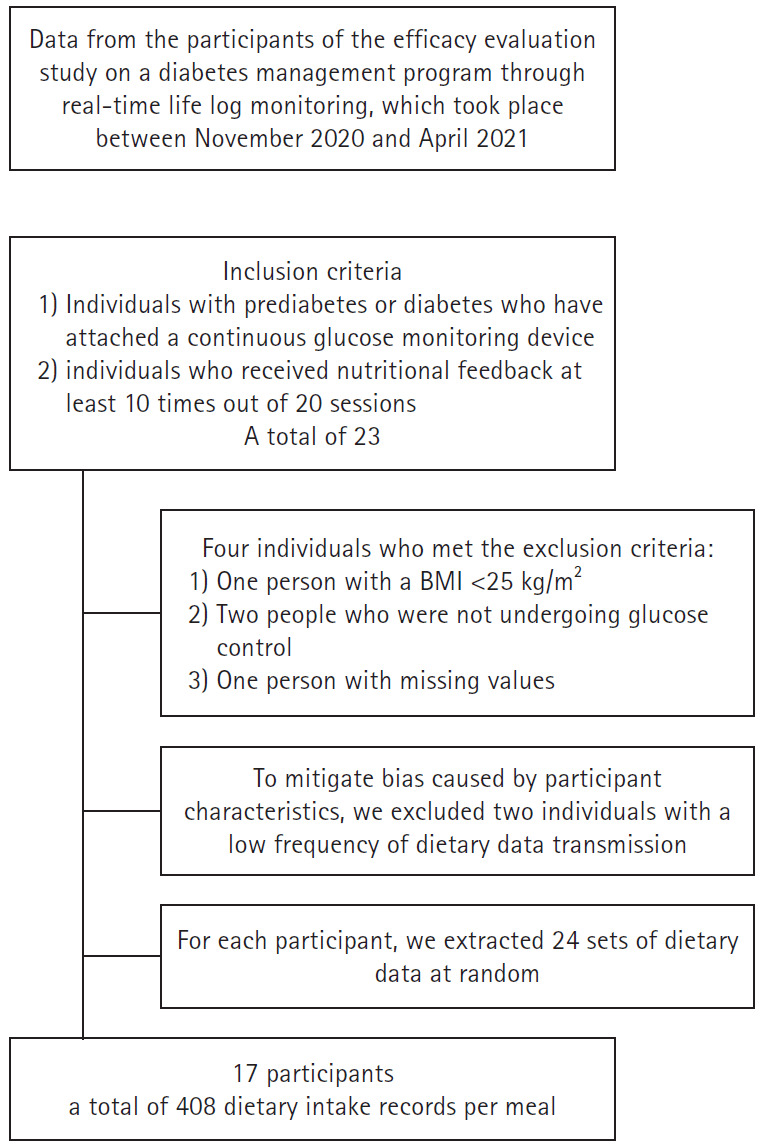
Flowchart of study population selection. BMI = Body mass index.

The inclusion criteria for the secondary analysis of this study were patients with prediabetes or diabetes who 1) wore the CGM for 4 weeks, 2) had a BMI ≥ 25, and 3) received at least 10 of 20 individualized dietary coaching using their diet photos. The exclusion criteria were as follows: a) not taking oral hypoglycemic agents, b) missing data on physical examination or blood tests, and c) not sending more than 20 out of 60 diet photos during the study period. In total, 17 participants were included in the study. The statistical power of this study may not be guaranteed due to the lack of participants’ data defining the relationships between insulin resistance, DII, and postprandial glycemic control. Therefore, to include more data, the dietary- days were analyzed rather than individual patients. A random sample of 24 meals was selected from each participant using random case selection. A total of 408 meals consumed by 17 participants were analyzed (Figure 1).

### 3. Instruments

#### 1) General and body measurement characteristics

The general characteristics recorded were sex, age, medical history, and history of drug use. Anthropometric characteristics were BMI and waist circumference measured at the initial screening before starting the program. Obesity was defined as a BMI ≥ 25kg/m^2^ [35].

#### 2) Blood biochemical characteristics

Blood biochemical properties, including HbA1c, homeostasis model assistance-insulin resistance (HOMA-IR), total cholesterol, and triglycerides, high-density lipoprotein cholesterol, and low-density lipoprotein cholesterol, were measured by blood tests after an 8-hour fasting period. HOMA-IR is considered normal in Republic of Korea at ≤ 2.53 [36].


$$\text{HOMA}-\text{IR}=\frac{\left\{\text{fasting glucose }\left(\frac{\text{mg}}{\text{dL}}\right)\times \text{fasiting insulin }\left(\frac{\text{μU}}{\text{mL}}\right)\right\}}{405}$$

#### 3) Energy-adjusted DII (E-DII)

The E-DII is one of the dietary inflammatory indices and a tool that measures potential inflammatory effects of an individual's diet [28]. The E-DII was calculated using the intake of 31 nutrients included in the nutrition assessment program Computer Aided Nutrition analysis program (CAN-Pro 5.0) (Korean Nutrition Society, Seoul, Korea). Calculated dietary information was quantified per 1000 kcal based on the Korean mean and standard deviation intakes for each dietary parameter, which can increase nutrient density, as described by Hébert et al. [37]. Each meal intake was multiplied by 3 to meet the daily intake and then adjusted to 1000 kcal. The E-DII scores potentially range from -3 to +3, with negative scores indicating anti-inflammatory effects and positive scores indicating a pro-inflammatory effect.

#### 4) Peak postprandial glucose

CGM data from participants wearing the Freestyle Libre (Abbott Diabetes Care Inc., Alameda, CA, USA) were used in this study. To identify glucose levels related to meals, data were selected from the CGM based on the time of meal initiation, specifically peak postprandial glucose, pre-prandial glucose, and glucose levels at 1, 2, and 3 hours after starting a meal.

### 4. Data analysis

Data were analyzed using SPSS version 28 (IBM Corp., Armonk, NY, USA). Kolmogorov-Smirnov and Shapiro-Wilk tests were performed to test the normal distribution of the data. Risk factor variables were assessed for sphericity using Mauchly’s test. Sphericity refers to the equality of variances in the differences between all possible pairs of within-subject conditions. For an analysis of variance, it is essential that the relationships between all-time points remain consistent. Descriptive statistics were used to summarize variables. Spearman's rank correlation coefficient was used to examine the correlations between BMI, insulin resistance, E-DII, and postprandial glucose response. Since the data processing method used in this study may have potentially introduced bias by reflecting the data from 17 people repeatedly, the bootstrap technique, which involves resampling 5,000 times, was chosen. Hayes' SPSS PROCESS macro version 4.0 [38] Model 2 was applied to analyze the multiple mediation effects of insulin resistance and E-DII on the impact of BMI on peak postprandial glucose levels. A sample size of 5000 was specified for the bootstrapping test.

### 5. Ethical considerations

This study was conducted after approval from the Institutional Review Board of the Pukyong National University (IRB No. 1041386-202211-HR-71-02). When recruiting participants, we provided sufficient information about the purpose of the study, the voluntary nature of participation, and confidentiality of information. Subsequently, written informed consent was obtained from each voluntary participant.

## RESULTS

### 1. General characteristics

The average fasting blood sugar was 135.29 ± 29.29, with an HbAlc level of 6.91 ± 1.15 and insulin resistance (HOMA-IR) of 4.10 ± 2.64. The average E-DII score for each participant was -0.04 ± 1.12, and the average maximum glucose level after meals was 201.42 ± 42.66. Furthermore, the average daily glucose level was confirmed to be 139.16 ± 35.72 (Table 1).

### 2. Correlation between BMI, insulin resistance, E-DII score, and postprandial glucose

Insulin resistance was positively correlated with the peak postprandial glucose level (rho = .30, *p* < .001). E-DII score positively correlated with glucose levels 1 hour after meals (rho = .11, *p* = .022) and peak postprandial glucose (rho = .14, *p* = .004). Additionally, a strong positive correlation was found between the glucose levels 1 hour after meals and peak postprandial glucose level (rho = .86, *p* < .001) (Table 2).

### 3. Multiple additive moderation effects of insulin resistance and E-DII score

The impact of BMI on the peak postprandial glucose level was moderated by the E-DII score and insulin resistance, and the explanatory power of the model was 21.6%. First, the conditional effect of BMI, insulin resistance, and E-DII on peak postprandial glucose showed statistically significant relationships (B = −53.05, *p* < .001; B = −13.95, *p* = .064; B = 15.18, *p* < .001, respectively). Additionally, the interaction between BMI and insulin resistance (B = 12.51, *p* = .002) and the interaction between BMI and E-DII score (B = −8.30, *p* < .001) were found to be statistically significant. Therefore, the impact of insulin resistance on the relationship between BMI and peak postprandial glucose changes depended on the E-DII score (Table 3).

**Figure 2.**
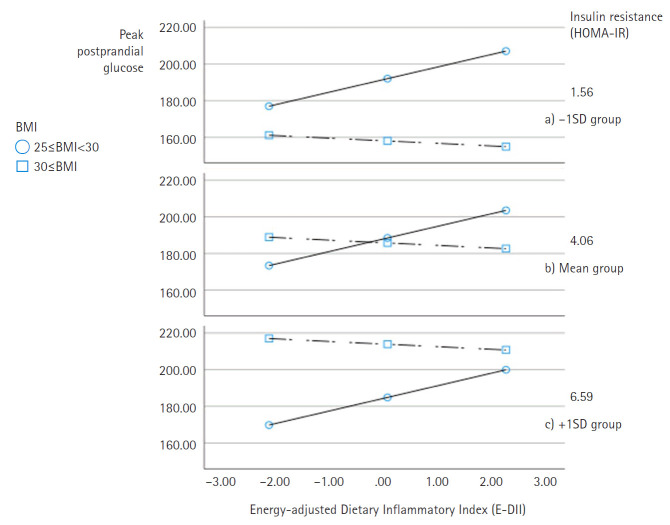
Visual representation of the moderating effect of insulin resistance and E-DII on the relationship between BMI and peak postprandial glucose. BMI = Body mass index; HOMA-IR = Homeostasis model assessment-insulin resistance; SD = Standard deviation. *This figure illustrates the relationship between E-DII and peak postprandial glucose, stratified based on HOMA-IR levels (-1SD, Mean, +1SD). a) HOMA-IR 1.56 (–1SD): 25 ≤ BMI < 30: Peak postprandial glucose increased ~30 mg/dL (178→ 210 mg/dL) with higher E-DII. BMI ≥30: Peak postprandial glucose decreased ~10 mg/dL (160→150 mg/dL) with higher E-DII. b) HOMA-IR 4.06 (Mean): 25 ≤ BMI < 30: Peak postprandial glucose increased ~30 mg/dL (170→200 mg/dL) with higher E-DII. BMI ≥ 30: Peak postprandial glucose decreased ~10 mg/dL (190→180 mg/dL) with higher E-DII. c) HOMA-IR 6.59 (+1SD): 25 ≤ BMI < 30: Peak postprandial glucose increased ~30 mg/dL (170→200 mg/dL) with higher E-DII.BMI ≥ 30: Peak postprandial glucose decreased ~10 mg/dL (220→210 mg/dL) with higher E-DII.

Figure 2 shows that peak postprandial glucose levels in patients with mild obesity or overweight (25 ≤ BMI < 30) increased with the E-DII score, and those in patients with obesity (BMI ≥ 30) decreased as the E-DII score increased, regardless of the insulin resistance level. However, for obese patients, when examining the differences according to insulin resistance level, for those with a mean insulin resistance level of 1.56, the range of postprandial glucose was between 150 and 160, whereas for those with a mean insulin resistance level of 6.59, the range of postprandial glucose started at 220, indicating a greater range of variability at higher levels of insulin resistance. HOMA-IR was not treated as a categorical variable based on the 2.53 threshold. Thus, for interpretation purposes, the participants were divided into three groups based on HOMAR-IR values: 1.56 (−1 standard deviation), 4.06 (mean), and 6.59 (+1 standard deviation). This approach allowed for a more detailed reflection of the subtle differences in the distribution of HOMA-IR values and degree of insulin resistance, and it enhanced statistical power when comparing groups.

## DISCUSSION

This study aimed to provide basic patient data for nursing interventions in overweight and obese patients by identifying the role of insulin resistance and E-DII in the relationship between BMI and postprandial glucose levels. Insulin resistance and E-DII score had multiple additive moderating effects on the relationship between BMI and peak postprandial glucose level.

The correlation analysis showed significant positive relationships between BMI, insulin resistance, and E-DII. The result showed that consuming foods with a high DII score was correlated with the risk of being overweight. Therefore, managing obesity requires recognizing the potential meals that cause inflammation and the dietary interventions that help people choose food with lower DII scores. The anti-inflammatory diet is not clearly defined because the E-DII score encompasses not only macro- and micro-nutrients but also bioactive components. Consequently, the finding of no relationship between insulin resistance and the E-DII in this study is difficult to interpret. However, consuming 10 g of dietary fiber per day for 8 weeks can improve fasting blood glucose and insulin resistance [39]. A 16-week vegetable-based dietary intervention also resulted in significant improvements in insulin resistance and beta-cell function in adolescents with obesity [40,41]. Although a vegetable-based diet contains many nutrients that can remove harmful reactive oxygen species and activate anti-inflammatory effects, it can lead to deficiencies in specific nutrients, such as protein, vitamins B, iron, zinc, and omega-3 fatty acids [42]. Further research is needed to stipulate an anti-inflammation diet and whether consuming a lower-DII score diet can help improve insulin resistance.

The E-DII score and insulin resistance were significantly related to the glucose level 1 hour after meals and the peak postprandial glucose level. The effect of E-DII scores on glucose levels may be more pronounced at 1 hour because glucose levels are typically highest during the first hour after eating [16]. Insulin resistance can lead to increased postprandial glucose levels if cells become less responsive to insulin signals after a meal, resulting in compensatory insulin secretion from pancreatic beta cells, which may eventually lead to impaired beta cell function. A cohort study conducted in Republic of Korea showed a strong correlation between postprandial glucose levels at 1 hour and beta cell dysfunction, with a cutoff value of 145mg/dL indicating a 2.84-fold higher risk of developing diabetes [43]. Other studies suggested that a postprandial glucose level of 155 mg/dL is a powerful predictor of diabetes [25,44]. Since peak postprandial glucose levels are measured 1 hour after starting a meal, further research is necessary to examine the relationship between peak postprandial glucose levels, insulin resistance, and beta cell dysfunction.

Insulin resistance and E-DII score showed different multiple moderating effects depending on the BMI group. In patients with mild obesity or overweight, peak postprandial glucose levels increased in response to elevated meal-induced inflammation regardless of the insulin resistance level. This result is supported by a previous study showing that higher DII scores were associated with higher glucose levels 2 hours after a 75 g oral glucose load, thus individuals with higher DII scores, indicating a more pro-inflammatory diet, tended to have higher glucose levels after the glucose load [29]. These findings support the notion that postprandial inflammation induced by mixed meals is associated with the postprandial glucose response, which in turn is associated with the inflammation and development of T2D. Moreover, adults who consume a more inflammatory diet have a higher risk of progression to prediabetes, development of T2D, and severity of diabetes than those who consume a non-inflammatory diet [30,45,46].

For patients with obesity, their postprandial glucose levels decreased as their E-DII score increased, whereas the range of postprandial glucose levels varied depending on their insulin resistance level. This finding is believed to be a consequence of participants characteristics because all patients with obesity received insulin therapy. Although this study did not control for dosage or frequency of insulin administration, the variation in postprandial glucose levels according to insulin resistance was significant. Insulin therapy is commonly used to treat T2D and rapidly lowers glucose levels, however, insulin resistance poses challenges in effectively regulating postprandial glucose levels using insulin therapy alone [47]. It showed the importance of interventions to regulate obesity indices and insulin resistance to reduce postprandial glucose levels. Therefore, it is crucial to consult health professionals, evaluate lifestyle habits, and continuously monitor glucose responses to determine the appropriate type and amount of insulin to improve lifestyle habits. The participants with T2D were registered in a dietary coaching program and may have exercised after consuming unhealthy foods to regulate their glucose levels. In general, patients treated with basal insulin have lower glycemic variability, indicating that blood glucose levels gradually increase after food intake [48]. Therefore, it is important to monitor the maintenance of glucose levels within an appropriate range throughout the day to improve glucose regulation in participants receiving insulin.

Based on the results of this study, healthcare providers can guide patients to adopt diets with lower E-DII scores, such as those rich in dietary fiber and vegetables, which have been shown to improve fasting glucose and insulin resistance. Nurses can play a vital role in educating patients about identifying inflammatory foods and making healthier dietary choices while ensuring nutritional adequacy to prevent deficiencies. Additionally, nurses can monitor patients’ postprandial glucose levels, provide personalized lifestyle coaching, and collaborate with interdisciplinary teams to tailor insulin therapy based on individual insulin resistance levels. CGM and regular assessments of BMI and dietary habits enable healthcare providers to adjust interventions, improve glycemic control, and prevent the progression of prediabetes and T2D.

### Limitations and future directions

This study has several limitations that need to be considered when interpreting the results. First, the study began after the intervention was conducted, limiting the number of participants available for data collection. Therefore, generalizability is limited. Future research should expand the scope of this study by recruiting larger numbers of participants or using a prospective longitudinal study design. Second, original scores may have been overestimated during the DII score adjustment process. The data were adjusted by considering previous studies that showed a threefold difference between daily nutrient intake and meal-specific intake [49,50]. This emphasizes the necessity of conducting future studies on meal-specific intake. Finally, while the meal-specific dietary intake data was collected repeatedly for each participant, the statistical analysis was conducted on individual independent samples. This approach assumed independence among the 408 individuals without considering potential individual random effects. Therefore, to improve statistical analysis accuracy, it is recommended that future researchers use a multilevel analysis to account for within-subject correlations in repeated measurements.

## CONCLUSION

This study found that insulin resistance and the E-DII significantly influenced the relationship between BMI and postprandial glucose levels. A pro-inflammatory diet, reflected by higher E-DII scores, was linked to elevated glucose levels, particularly in individuals who were overweight and mildly obese. In patients with obesity undergoing insulin therapy, glucose variability was more affected by insulin resistance than by dietary inflammation. These results emphasize the need for personalized interventions that address both dietary inflammation and insulin sensitivity. CGM and tailored lifestyle modifications are essential for effective obesity and glucose management.

## Figures and Tables

**Table 1.** Demographics and Clinical Characteristics of the Participants (N = 17)

Characteristics	Categories	n (%)	M ± SD
Sex	Men	5 (29.4)	-
	Women	12 (70.6)	-
Age (yr)		-	48.0 ± 11.82
Abdomen circumference (cm)		-	95.08 ± 7.61
BMI (kg/m^2^)		-	28.48 ± 3.84
	25-29	9 (52.9)	25.57 ± 2.62
	≥ 30	8 (47.1)	31.76 ± 1.60
Fasting glucose (mg/dL)		-	135.29 ± 29.29
HbAlc (%)		-	6.91 ± 1.15
	< 6.5	8 (47.1)	5.98 ± 0.47
	≥ 6.5	9 (52.9)	7.86 ± 0.77
Insulin resistance (HOMA-IR)		-	4.10 ± 2.64
	< 2.5	6 (35.3)	1.99 ± 0.40
	≥ 2.5	11 (64.7)	5.24 ± 2.63
Total cholesterol		-	147.65 ± 40.98
Triglyceride		-	151.35 ± 61.34
HDL		-	48.32 ± 11.21
LDL		-	81.18 ± 30.62
Comorbidities	Hypertension	2 (11.8)	-
	Dyslipidemia	7 (41.2)	-
	Both	3 (17.6)	-
	None	5 (29.4)	-
Diabetes diagnosis	Prediabetes	7 (41.2)	-
	Type 2 diabetes	10 (58.8)	-
Treatment	OHA	9 (52.9)	-
	OHA and insulin	8 (47.1)	-
E-DII		-	−0.04 ± 1.12
Peak postprandial glucose according to each meal		-	201.42 ± 42.66
Mean average glucose per day		-	139.16 ± 35.72

M = Mean; SD = Standard deviation; BMI = Body mass index; HbA1c = Glycated hemoglobin; HOMA-IR = Homeostasis model assessment-insulin resistance; HDL = High-density lipoprotein; LDL = Low-density lipoprotein; OHA = Oral hypoglycemic agent; E-DII = Energy adjusted dietary inflammatory index.

**Table 2.** Correlations between BMI, Insulin Resistance, E-DII, and Postprandial Glucose Levels (N = 408)

	BMI	Insulin resistance	E-DII	1h PG	2h PG	3h PG	Peak PG
	r(*p*)						
Insulin resistance	.76 (< .001)	1	-	-	-	-	-
E-DII	.24 (< .001)	.08 (.090)	1	-	-	-	-
1h PG	.09 (.068)	.22 (< .001)	.11 (.022)	1	-	-	-
2h PG	.11 (.024)	.37 (< .001)	.08 (.110)	.69 (< .001)	1	-	-
3h PG	.22 (< .001)	.44 (< .001)	.10 (.051)	.43 (< .001)	.77 (< .001)	1	-
Peak PG	.09 (.080)	.30 (< .001)	.14 (.004)	.86 (< .001)	.85 (< .001)	.63 (< .001)	1

BMI = Body mass index; E-DII = Energy adjusted dietary inflammatory index; PG = Postprandial glucose; h = hour.

**Table 3.** Multiple Additive Moderating Effects of Insulin Resistance and E-DII on the Relationship between BMI and Peak Postprandial Glucose (N = 408)

		B	SE	t(*p*)	R^2^	Δ R^2^	F(p)
(Constant)		246.95	21.28	11.60 (< .001)	.22		
Independent variable	BMI (Ref. : < 30)	−53.05	12.73	−4.17 (< .001)			
Moderation variable	Insulin resistance	−13.95	7.50	−1.86 (.064)			
Moderation variable	E-DII	15.18	3.33	4.56 (< .001)			
BMI ×Insulin resistance		12.51	3.91	3.20 (.002)		.02	10.26 (.002)
BMI × E-DII		−8.30	2.13	−3.90 (< .001)		.03	15.22 (< .001)
Both						.05	12.22 (< .001)

E-DII = Energy adjusted dietary inflammatory index; BMI = Body mass index; SE = Standard error; Ref = Reference.

## Data Availability

The data that support the findings of this study are available from the corresponding author upon reasonable request
